# Reply to Prueitt and Goodman, “Real-world bisphenol A exposure not linked to microbiota dynamics in childhood obesity”

**DOI:** 10.1128/msystems.00714-24

**Published:** 2024-07-03

**Authors:** Ana López-Moreno, Margarita Aguilera, Alicia Ruiz-Rodríguez

**Affiliations:** 1 Department of Microbiology, Faculty of Pharmacy, University of Granada, Campus of Cartuja, Granada, Spain; 2 Institute of Nutrition and Food Technology "José Mataix" (INYTA), Centre of Biomedical Research, University of Granada, Granada, Spain; 3 Department of Biochemistry and Molecular Biology II, Faculty of Pharmacy, Campus of Cartuja, University of Granada, Granada, Spain; University of California, San Diego, La Jolla, California, USA

**Keywords:** exposure, gut microbiota, obesity, bisphenol A (BPA)

## REPLY

We acknowledge the discussion on the complexity for evaluating the real impact of bisphenol A (BPA) human exposure (1). Our paper titled “Bisphenol A exposure affects specific gut taxa and drives microbiota dynamics in childhood obesity” showed as main findings that gut microbiota taxa with tolerance to BPA were differentially represented among children with different body mass index (obese and normal weight) and how these microbial taxa affected the overall dynamics toward patterns of diversity generally recognized in microbiota dysbiosis.

The study was designed as a pilot exploration into how BPA exposure might select taxa and influence gut microbiota diversity. While the BPA concentrations used would not exhaustively reflect real human exposure, our *in vitro* study did not intend to directly replicate *in vivo* conditions and effects, but only to assess the toxicological impact on gut microbiota.

The final BPA concentrations chosen were 20 ppm and 50 ppm, aiming to enhance the selection of resilient, tolerant versus susceptible taxa and identify potential microbial shifts under BPA-controlled conditions. Further (BPA) calculations or equivalence does not proceed according to the aim of the experimental procedure (where, in any case, calculated correspondence to 250 mg of BPA does not proceed). Furthermore, regulation of the future is envisaging to restrict plastic mixture containing bisphenols in concentrations higher than 10 ppm (0.001% by weight).

Moreover, scientific evidence based on BPA biomonitoring and extensive toxicological data gathered during the last decade allowed for the re-establishment of a reduced BPA tolerable dietary intake (TDI) of 0.2 ng/kg body weight per day, which was 20,000 times lower than the previous TDI of 4 µg/kg body weight per day (2). However, BPA dietary exposure estimations (3, 4) and experimental studies (5) were higher than new TDI. Additionally, it is important to highlight that cumulative exposure to BPA, analogs, and similar chemical plasticizers cannot be neglected (6, 7), but summed up, and therefore, it would largely increase the exceedance of the TDIs.

Importantly, the processes of bioaccumulation, metabolization, and excretion of BPA and analogs are not fully understood; consequently, several routes could not be excluded (8). Moreover, data based on urine biomonitoring for evaluating human exposure is under discussion, as the presence of BPA in urine does not have the same meaning as its presence elsewhere in the body. BPA and its analogs could be metabolized very rapidly in the body (9); therefore, single spot urine samples have limited ability to reflect long-term exposure levels (10). Other biospecimens (such as nail, hair, etc.) have been proposed for robust assessments, as they experienced less fluctuations in concentration levels (5). Consequently, equivalence of children’s BPA exposure cannot be extrapolated based solely on urine levels.

We acknowledge the controversial nature of BPA exposure and metabolization data in obese populations compared to non-obese populations. Nonetheless, over the last 5 years, several studies have aimed to demonstrate the obesogenic effect of BPA exposure (11–13). However, the primary aim of our study was not to demonstrate impairment of BPA metabolism in obese children but, rather, to show differential capabilities of the gut microbiota in obese and normal-weight children in response to BPA.

In conclusion, we recognize the challenges inherent in extrapolating *in vitro* findings to real-world human exposure scenarios. Our study, designed to generate hypotheses, explored the potential effects of BPA on gut microbiota dynamics in children with obesity and non-obesity. It does not posit direct causal links between typical human BPA exposure levels and microbiota changes associated with obesity. Rather, our research explored microbial responses under controlled experimental conditions. Going forward, our findings advocate for future studies integrating microbiota into the whole pharmacokinetics of BPA.
